# TNFRSF11B modulates Marek’s disease virus infection by regulating apoptosis in chicken embryo fibroblasts

**DOI:** 10.3389/fvets.2026.1727743

**Published:** 2026-02-05

**Authors:** Gang Zheng, Yuqin Yang, Yushuang Du, Yongzhen Liu, Ling Lian

**Affiliations:** 1 Frontiers Science Center for Molecular Design Breeding, China Agricultural University, Beijing, China; 2 State Key Laboratory of Farm Animal Biotech Breeding, China Agricultural University, Beijing, China; 3 National Key Laboratory for Animal Disease Prevention and Control, Chinese Academy of Agricultural Sciences, Harbin Veterinary Research Institute, Harbin, Heilongjiang, China

**Keywords:** chicken, Marek’s disease virus, Poly I:C, TNFRSF11B, apoptosis

## Abstract

**Introduction:**

Marek’s disease, caused by the oncogenic Marek’s disease virus (MDV), remains a major threat to poultry production. Host immune responses and apoptosis pathways play critical roles in MDV pathogenesis, yet the underlying mechanisms are not fully understood.

**Methods:**

Chicken embryo fibroblasts (CEF) were treated with poly I:C to assess its effect on MDV infection, followed by transcriptome profiling to identify differentially expressed genes. Functional assays using siRNA knockdown and overexpression of TNFRSF11B were performed to evaluate its impact on viral infection and apoptosis.

**Results:**

Poly I:C treatment significantly reduced MDV infection in CEF. Transcriptome analysis identified 274 DEGs, among which TNFRSF11B was notably downregulated. Consistently, MDV infection suppressed TNFRSF11B expression in both CEF and spleen tissues. Functional assays revealed that knockdown of TNFRSF11B enhanced MDV infection, whereas its overexpression suppressed MDV infection. Furthermore, MDV infection induced substantial cell apoptosis, and TNFRSF11B knockdown further exacerbated this effect, as demonstrated by elevated CASP3 expression and higher apoptosis rates. A strong positive correlation was observed between MDV infection levels and apoptosis rates (*R*^2^ = 0.9895, *p* < 0.0001).

**Discussion:**

Our findings indicated TNFRSF11B could modulate MDV infection through collaborating with MDV to modulate cell apoptosis. This study provides new insights into the pathogenesis of MDV and potential antiviral strategies targeting MDV/TNFRSF11b—cell apoptosis.

## 1 Introduction

Marek’s disease (MD) is a neoplastic disease caused by Marek’s disease virus (MDV), leading to immunosuppression in susceptible chickens, which poses a serious threat to chicken production (1). Currently, the primary approach for controlling this disease is vaccination (2). However, vaccination drives the evolution of viral virulence toward increased strength (3–5). A key viral oncogene, meq, was positively selected in modern strains (6), has undergone ordered loss of tetraproline repeats and increased transactivation ability. This may be one of the key reasons that have driven the evolution of MDV toward higher pathogenicity. Therefore, it is essential to investigate the interaction mechanisms of MDV and hose in order to improve preventive measures in poultry production.

MDV, as a cell-associated virus, is dependent on cells for its survival. Chicken Embryo Fibroblasts (CEF) from SPF (Specific Pathogen Free) chicken embryo have been widely used in the MD study, not only for the production of vaccines (7), but also acting as an important cell type infected by Marek’s disease virus (MDV) in MD study *in vitro* (8). In some infection assays (9, 10), Toll-like receptors (TLRs) as a receptor recognizing pathogen associated molecular patterns (PAMPs) have been suggested to be involved in MDV infection. It has been reported that treatment with double-stranded viral mimic polyinosinic: polycytidylic acid (poly I: C), a ligand of the TLR3, significantly reduced the infection level of the hypervirulent strain RB1B in CEF (9, 11). However, the precise molecular mechanisms underlying the antiviral response against MDV infection and whether CEF exhibit the same antiviral response when confronted with other viral strains remain unclear.

In this study, we corroborated the protective effect of poly I: C against MDV infection and we identified a gene, TNFRSF11B, downregulated in CEF post poly I: C stimulation. TNFRSF11B is a member of the TNF family, and its primary function is to inhibit osteoclast formation and activity (12). Meanwhile, TNFRSF11B has been reported in a variety of oncological diseases. Its expression is up-regulated in a variety of tumor tissues (13–15), mainly by acting as a decoy receptor for TRAIL, inhibiting apoptosis mediated by TRAIL, thereby sparing tumor cells from apoptosis. In human, it was reported that expression of TNFRSF11B was upregulated in persistent hepatitis C virus (HCV) infected individuals (16). However, to the best of our knowledge, no studies have investigated the association between TNFRSF11B and avian virus infection. In this study, we found TNFRSF11B was downregulated *in vivo* and *in vitro* during MDV infection, and for the first time, we investigated the association among TNFRSF11B, apoptosis, and MDV infection, and found that MDV infection significantly induced cell apoptosis and TNFRSF11B knockdown exacerbated this apoptosis level, which indicated TNFRSF11B collaborating with MDV, regulated MDV infection. This provides us with new clue for exploring the interactions between MDV and its host.

## 2 Materials and methods

### 2.1 Preparation of cells, viruses, and tissue samples

Seven-day-old chicken embryos were obtained from Boehringer Ingelheim International GmbH. Tissues after removal of the head, neck, limbs and viscera were minced, washed by Hanks buffer (GIBCO, United States), digested with 0.25% trypsin (GIBCO, United States), resuspended in DMEM medium (GIBCO, United States) containing 5% fetal bovine serum (FBS, GIBCO, United States) and 1% penicillin/streptomycin (PS, GIBCO, United States), filtered through a 40um mesh filter, and incubated in an incubator at 37 °C with 5% CO_2_. MDV recombinant fluorescent strain 814-GFP was obtained from the Harbin Institute of Veterinary Medicine, Chinese Academy of Agricultural Sciences, and the strain virulence was obtained by cascade dilution and plaque counting. MDV-infected spleen and normal spleen tissues from the previous challenge assay (17).

### 2.2 Treatment of poly I: C

The high dose (25 μg/ML) and low dose (2.5 μg/ML) poly I: C (Sigma-Aldrich, United States) were prepared, and diluent (sterile water) was used as a control. CEFs were cultured in 12-well plates, and then treated with high or low dose of poly I: C or diluent for 24 h. After treatment, the cells were divided into two parts, and a portion of the cells was used to extract RNA for RT-qPCR and subsequent transcriptome analysis. The other part of cells was subjected to 814-GFP infection (2,000 pfu per well), and the CEFs were collected at 48 h post infection, and used for flow cytometry experiment to detect the rate of infection.

### 2.3 Detection for MDV infection level by flow cytometry

The recombinant virus MDV 814-GFP was used for CEF infection, and the infected cells were collected at 48 or 72 h post infection, and then resuspended in pre-cooled DPBS (GIBCO, United States). GFP-positive cells were identified using flow cytometry (BD Biosciences, Mississauga, ON, Canada), and infection level were analyzed using Flowjo software (Tree Star, Ashland).

### 2.4 RT-qPCR

RNA was extracted using NucleoZOL (MACHEREY-NAGEL, Germany), and 2 μg RNA was used for reverse transcription using Fastking cDNA First Strand Synthesis Kit (TIANGEN, China). The cDNA obtained from reverse transcription was used for quantitative PCR using Talent Fluorescence Quantification Kit (TIANGEN, China) in BIO-RAD CFX96 (BIO-RAD, Singapore). The primers were synthesized by Beijing Liuhe BGI (China).

### 2.5 Copy number detection for MDV

DNA was extracted from the MDV infected cells by TIANamp Genomic DNA Kit following standard procedures provided by the manufacturer (TIANGEN, China). The primers and probes used are referred to previous study (18). Probe qPCR was conducted using 2 × T5 Fast dUTP/Heat Labile System Probe qPCR mix (Tsingke, China).

### 2.6 RNA library construction and sequencing

Cells treated with high poly I: C or diluent were collected for transcriptome sequencing as follows: total RNA was used for library construction after quality control, and Oligo (dT) magnetic beads were used to enrich mRNAs with polyA tails. The NEB NEXT Ultra TMRNA Library Prep kit (NEB, United States) was used for library construction, and then next-generation sequencing (NGS) was conducted on Illumina platforms, and the final output was 6 G of data per sample.

### 2.7 Knock down assay

The three siRNAs (Table 1) for TNFRSF11B were synthesized by Jiangsu Genecefe Biotechnology (China). Transfection of siRNAs was performed in 24-well plates (30 pmol per well) following siRNA-mate plus transfection kit procedure (GenePharma, China). The cells were collected at 24 h post transfection for RNA extraction to detect the interference efficiency, and the siRNAs with significant knockdown effect were further used for the following experiment.

**Table 1:** siRNA sequences for TNFRSF11B.

The name of siRNAs	Sense (5′–3′)	Antisense (5′–3′)
TNFRSF11B-411	GAGUGACACUGUUUGUAAATT	UUUACAAACAGUGUCACUCTT
TNFRSF11B-957	GUGUCAACAUGCAGAGCAATT	UUGCUCUGCAUGUUGACACTT
TNFRSF11B-789	GGAGCAGGAUAUGGUCAAATT	UUUGACCAUAUCCUGCUCCTT

### 2.8 Overexpression assay

The TNFRSF11B full mRNA sequence (1,299 bp) was obtained from the NCBI website and cloned into the pCDNA 3.1 (+) expression vector after adding a 3xFLAG tag sequence at the C-terminus, to construct pCDNA3.1-TNFRSF11B-3 × FLAG vector. The 2 μg vector was transfected into 1 × 10^6^ CEFs by electrotransfection, using the electrotransfection kit from LONZA (Swiss) according to the procedures provided.

### 2.9 Immunoblotting assay

Total protein was collected from treated cells, and protein concentration was determined using a BCA kit (Beyotime, China), with 20 μg of protein per well for electrophoresis. Primary antibodies used included: FLAG mouse antibody (1:5,000, ABMART, China), *β*-actin rabbit antibody (1:50,000, ABconal, China). Secondary antibodies used were goat anti-mouse/rabbit HRP-labeled antibodies (ABconal, China), respectively.

### 2.10 Apoptosis assay

The cells were pelleted by centrifugation, and then resuspend‌ by PBS. About 1 × 10^5^ cells were centrifuged and then Annexin V-mCherry Bingding Buffer was added to resuspend cells, followed by adding Annexin V-mCherry mixing and incubation for 10 min away from the light. The apoptosis rate was detected by flow cytometry following the standard procedure of the kit (Beyotime, China).

### 2.11 Data analysis

The quality control of data obtained from transcriptome sequencing was performed by fastp and then the reads were aligned to the reference genome (Gallus_gallus. GRCg6a) by HISAT. FPKM values were calculated using feature Counts to quantify gene expression levels, followed by differential expression analysis using DESeq2. Pathway and gene set enrichment analysis were done using the R software.

The data was analyzed using student’s *t*-test (Two comparison group) or one-way ANOVA (Three or more comparison groups) in SPSS version 19.0. *P* < 0.05 means statistically significant difference,*P <* 0.01 means statistically highly significant difference. Statistical graphs are plotted using GraphPad Prism version 9.3.

## 3 Results

### 3.1 Poly I: C pretreatment reduces MDV infection in CEFs

**Figure 1:**
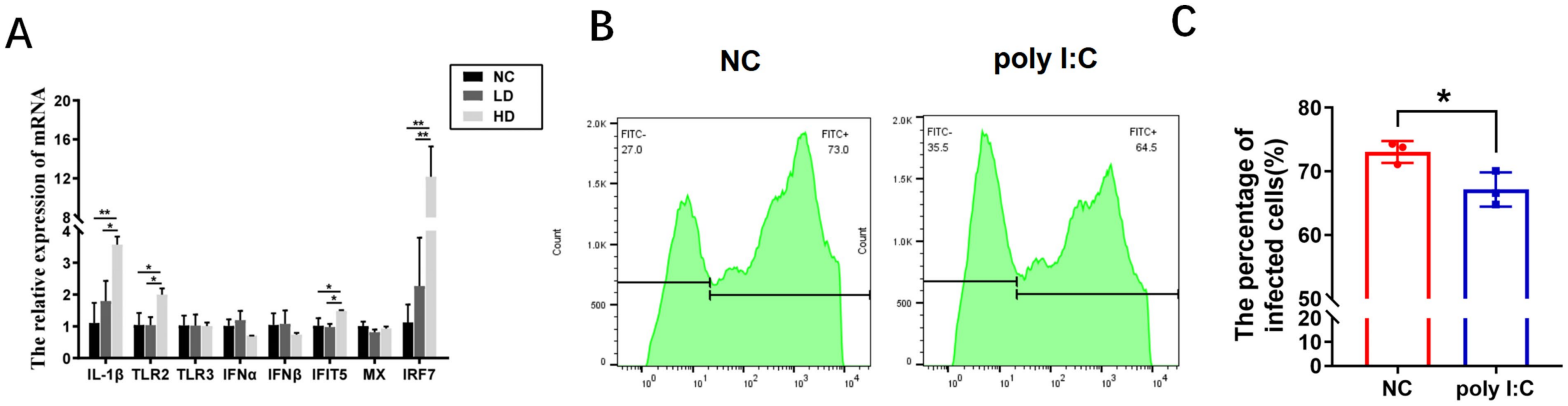
Poly I: C treatment activated the immune signaling pathway and downregulates MDV infection. **(A)** The expression of genes in TLR3 pathway among high-dose, low-dose, and control groups. RT-qPCR was used to detect the expression of genes related to the TLR3 signaling pathway with different doses of poly I: C pretreatment at 24 h post stimulation (HD means high dose and LD means low dose). Flow plots **(B)** and statistical graphs **(C)** of CEF stimulated by high-dose poly I: C for 24 h and then infected with 814-GFP for 72 h. Data is represented as mean ± SEM. Significant comparisons between two groups are made using Student’s *t*-test, and among three groups are made by one-way ANOVA (* means *p* < 0.05, and ** means *p* < 0.01).

The expression of cytokine genes IL-1*β*, IFN-*α*, TLR2, TLR3, IFN-β and interferon-stimulated genes IFIT5 and Mx as well as the interferon-regulated gene IRF7 were detected in CEFs treated with high and low doses of poly I: C for 24 h, and the results showed that the expression of interleukin genes IL-1β, IFIT5, IRF7, and TLR2 were significantly up-regulated in the high-dose group compared with the control group, and no significant expression differences for IFN-α, IFN-β, Mx, and TLR3 (Figure 1A). The results showed that the high-dose group elicited an immune response.

High-dose poly I: C was used to treat CEFs for 24 h followed by MDV 814-GFP infection, and the infection rate was detected by flow cytometry at 72 h post MDV infection. The results showed that poly I: C pretreatment significantly reduced the MDV infection rate (72.2%_vs_65.8%, *p* < 0.05) (Figures 1B,C).

### 3.2 Poly I: C and MDV treatment downregulate TNFRSF11B expression

**Figure 2:**
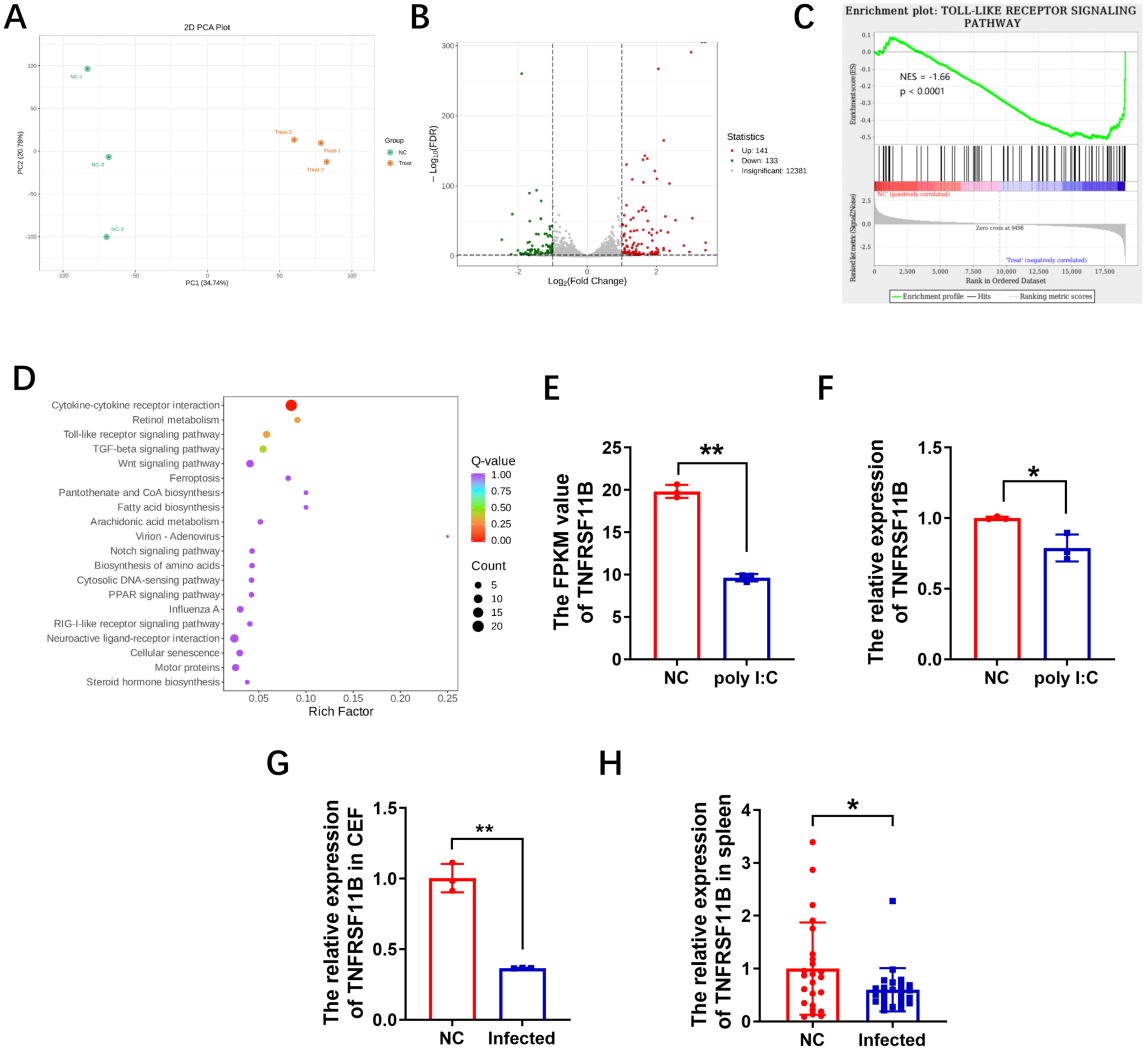
TNFRSF11B regulated MDV infection. **(A)** Knockdown of TNFRSF11B significantly reduced its expression. Flow plots **(B)** and statistical graphs **(C)** for 48 h of infection with 814-GFP after 24 h of TNFRSF11B knock-down. **(D)** Viral copy number detection after knockdown of TNFRSF11B. QPCR **(E)** and western blot **(F)** assays for overexpression of TNFRSF11B in CEF. Flow plots **(G)** and statistical graphs **(H)** of CEF infection rate when CEF was overexpressed with TNFRSF11B for 24 h and then infected with 814-GFP for 48 h. Data is represented as mean ± SEM. Significant comparisons between two groups are made using Student’s *t-t*est (* means *p* < 0.05, and ** means *p* < 0.01).

The CEFs treated with poly I: C high-dose group and controls were collected at 24 h post stimulation, and were subjected to transcriptome sequencing, and principal component analysis (PCA) revealed significant differences between treated and non-treated groups (Figure 2A). Differentially expressed genes (DEGs) were subsequently identified and plotted in a volcano plot, and a total of 274 differentially expressed genes were identified, of which 141 were down-regulated and 133 up-regulated in the poly I: C treatment group compared with control group (Figure 2B; Supplementary Table S1). Gene set enrichment analysis (GSEA) revealed that Toll-like receptor signaling pathway was one of pathways that DEGs were significantly enriched in Figure 2C. Pathway enrichment analysis revealed that cytokine interaction with its receptor was the most significantly enriched pathway that DEGs enriched in Figure 2D, involving 20 genes, of which 17 were up-regulated and 3 down-regulated in the poly I: C treatment group compared to control group. These genes are concentrated in four categories: chemokines-related genes, interleukin-related gene, TNF family genes, and TGF-*β* family genes (Supplementary Figure S1). Three of the downregulated genes were TNFRSF11B, ENSGALG00000012055, and GDF9. Among these three downregulated genes, TNFRSF11B has been extensively studied in a variety of oncological diseases, but has been rarely reported in MD study. Therefore, we selected this gene for further study. The expression of TNFRSF11B was verified by qPCR, and the results were consistent with the trend of RNA sequencing results (Figures 2E,F). To further analyze the function of TNFRSF11B in MDV infection, we examined the expression of TNFRSF11B in CEF at 48 h post MDV infection and found that expression of TNFRSF11B was down-regulated (Figure 2G). At the same time, we also compared TNFRSF11B expression in MDV-infected and non-infected spleens, and the results also showed that TNFRSF11B expression in MDV-infected spleen was also lower than that in non-infected spleens (Figure 2H).

### 3.3 Knockdown TNFRSF11B promotes MDV infection

**Figure 3:**
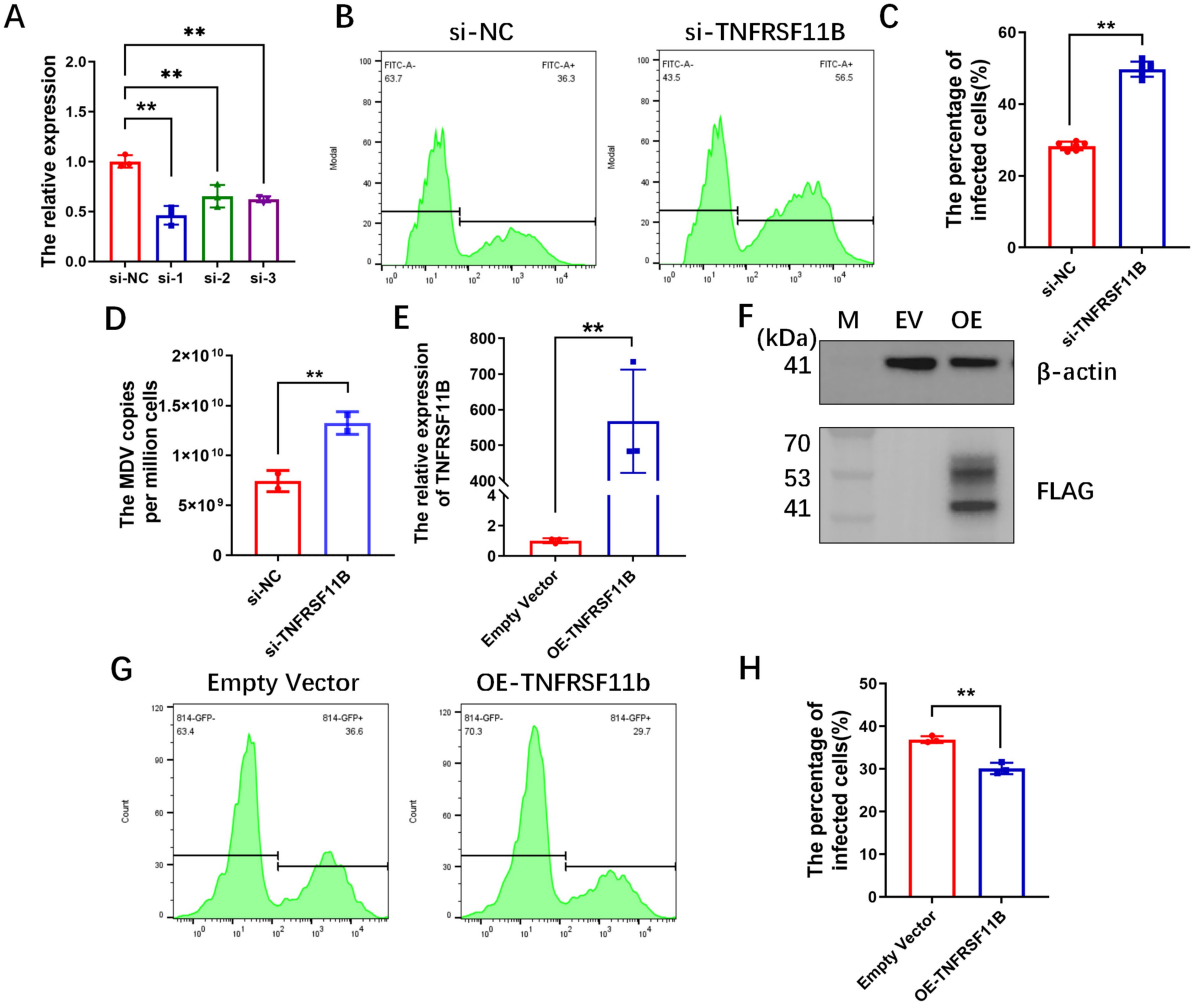
Transcriptome sequencing analysis of poly I: C pretreated CEFs after 24 h. **(A)** PCA analysis demonstrating significant differences between poly I: C treatment and non-treatment groups. **(B)** Volcano diagram showing the identified differentially expressed genes (DEGs). **(C)** Gene set analysis (GSEA) highlighting the TLR receptor signaling pathway in the poly I: C treatment group. **(D)** KEGG enrichment analysis demonstrates that the cytokine-cytokine receptor interaction pathway is significantly enriched. Transcriptome sequencing **(E)** and RT-qPCR results **(F)** showed that TNFRSF11B expression was downregulated in the poly I: C treated group. QPCR results of TNFRSF11B in MDV-infected cells **(G)** and tumor spleens (*N* = 24_vs_24) **(H)**. Data are represented as mean ± SEM. Significant comparisons between two groups are made using Student’s *t-*test (* means *p* < 0.05, and ** means *p* < 0.01).

To verify the effect of TNFRSF11B on MDV infection, we performed loss-of-function assay. At first, a total of three interfering fragments were synthesized for TNFRSF11B and transfected into CEF, and the knockdown efficiency was assessed by RT-qPCR at 24 h post transfection, which showed that all the three interfering fragments had significant knockdown effect (Figure 3A). The siRNA with the highest interference efficiency was selected. The CEF was infected with 814-GFP at 24 h post siRNA treated and infection rate of CEF were detected by the flow cytometry at 48 h post infection. The results showed a significant increase (36.03%_vs_56.82%) in MDV infection after knockdown of TNFRSF11B (Figures 3B,C). Detection of viral copy number was also increase by 78.19% (7.44 × 10^9^_vs_1.33 × 10^10^ per million cells) (Figure 3D).

### 3.4 Overexpression of TNFRSF11B inhibits MDV infection

Subsequently, we performed gain-of-function experiments, the overexpression vector pCDNA 3.1-TNFRSF11B-3 × FLAG and the control vector pCDNA 3.1–3 × FLAG were transfected into CEF, respectively. The cells were collected for qPCR and western blot assay at 48 h post transfection, and the results showed TNFRSF11B was effectively overexpressed at both RNA (Figure 3E) and protein levels (Figure 3F). After confirming the validity of the expression, subsequent cell infection experiments showed a significant decrease of MDV infection at 48 h post overexpression of TNFRSF11B (36.90%_vs_30.10%) (Figures 3G,H).

### 3.5 The knockdown of TNFRSF11B promotes apoptosis induced by MDV in infected cells, increasing MDV infection level

**Figure 4:**
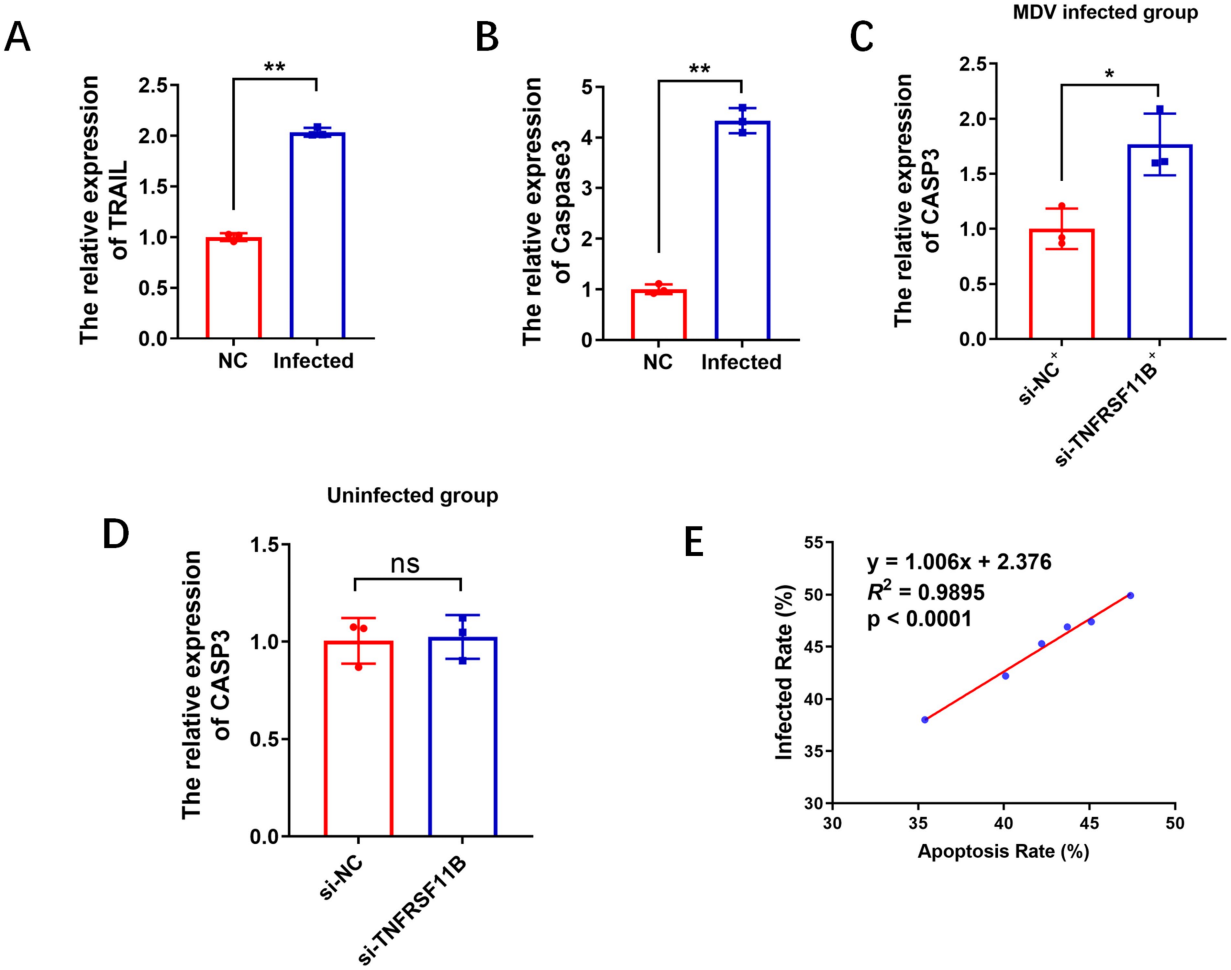
TNFRSF11B promotes apoptosis in MDV-infected cells. QPCR results of TRAIL **(A)** and CASP3 **(B)** in MDV-infected cells. MDV^+^ means MDV infection, and NC means negative control. **(C)** MDV infection was performed after TNFRSF11B knockdown and qPCR was performed to detect CASP3 expression. **(D)** CASP3 expression detected by qPCR after TNFRSF11B knockdown in non-infected cells. **(E)** Regression curves between apoptosis rate and infection rate. Data are represented as mean ± SEM. Significant comparisons between two groups are made using Student’s *t*-test (* means *p* < 0.05, and ** means *p* < 0.01).

Given that TNFRSF11B is closely related to the apoptosis signaling pathway and MDV infection can induce apoptosis, to further analyze the function of TNFRSF11B in this process, we firstly examined the expression of apoptosis-associated key genes TRAIL (Figure 4A) and CASP3 (Figure 4B) after MDV infection in CEF, and we found that the expression of these two genes was significantly up-regulated, and these results suggested that MDV induced cell apoptosis. To investigate whether TNFRSF11B involved in MDV-induced apoptosis, TNFRSF11B was knockdown in MDV infected cells and uninfected cells, and the results showed expression of CASP3 was up-regulated significantly in MDV-infected cells (Figure 4C), and meanwhile, the apoptosis rate of cells in knockdown group was higher than control group (Supplementary Figure S2A). However, the expression of CASP3 in non-MDV infected CEFs had no significant difference post TNFRSF11B knockdown (Figure 4D), which suggested the apoptosis could not be induced when cells with TNFRSF11B knockdown but without MDV infection, and knockdown of TNFRSF11B promoted apoptosis induced by MDV. In further, we detected the infection rate at 24 h post MDV infection following si-TNFRSF11B transfection and found that the MDV infection level in the knockdown group was significantly higher than that in the control group (15.6%_vs_24.5%) (Supplementary Figure S2B). To further explore the relationship between apoptosis and infection, we analyzed the rate of apoptosis and the rate of infection and the results showed that apoptosis rate and the infection rate have a strong positive correlation (*R*^2^ = 0.9895, *p* < 0.0001) (Figure 4E). These results suggested that the effect of MDV promoted apoptosis could be aggravated by TNFRSF11B knockdown, which in turn promoted further cellular infection *in vitro*.

## 4 Discussion

TLR ligands play a protective role as prophylactic agents in a variety of infectious diseases (19, 20), with poly I: C found to be the most effective in inhibiting MDV hypervirulent strain RB1B infection in in vitro studies, however it is not yet known whether it is effective across different MDV strains. In this study, we used the weak recombinant strain of MDV, 814-GFP, and the results showed that poly I: C reducing MDV infection (73.8_vs_66.6% at 72 h post infection) was remain effective, but the decrease of infection level was not as strong as that after the RB1B strain infection (40.8% _vs_ 7.96% at 96 h post infection).

To investigate the mechanisms and genes involved in decreasing MDV infection level, we conducted transcriptome analysis between poly I: C-treated and untreated control cells, and we found TNFRSF11B was down-regulated post poly I: C stimulation. It was reported that TNFRSF11B is expressed in a variety of cancers, such as colorectal cancer (15), prostate cancer (21), gastric cancer (13), lung cancer (22), etc., and its expression usually higher in these cancer model, which inhibited TRAIL-induced apoptosis in the tumor cells, promoting the proliferation and metastasis of tumor cells. In some tumor tissues, TNFRSF11B has also been reported to be low-expressed and is often associated with poor prognosis or promotion of tumor growth, such as in breast cancer (23), myeloma (24, 25).

However, little research has been reported in chicken disease. In this study, we established an MDV-infected CEF model, and we found TNFRSF11B was down-regulated, which was also observed in MDV-infected spleens. We were wandering whether this down-regulation was one mechanism of host defense, so we knocked down TNFRSF11B in CEF. Surprisely, the MDV infection was further promoted, while TNFRSF11B overexpression reduced MDV infection level. Subsequent assay showed that apoptosis induced by MDV could be further enhanced by knocking down TNFRSF11B *in vitro*, and a strong positive correlation exhibited between infection and apoptosis rates. In summary, there is a positive feedback between in vitro MDV infection and cell apoptosis, and TNFRSF11B showed an apoptosis-promoting effect during this process, which suggested that MDV might utilize this mechanism to sustain infection and facilitate transmission.

Actually, utilization of apoptosis by tumor cells to achieve immune escape were previously reported, e.g., CD70-mediated apoptosis of lymphocytes in renal cell carcinoma (RCC) (26). CD70 is a cytokine that is overexpressed in RCC and promotes lymphocyte apoptosis through interaction with its receptor CD27 and the intracellular receptor binding protein SIVA. This may further result in the inability to generate an effective lymphocyte-mediated antitumor response. African swine fever virus (ASFV) could induce apoptosis and spread the virus through apoptotic body as one of the ways of viral infection (27). MERS-CoV infection has been reported (28) to activate RNA-like endoplasmic reticulum protein kinase (PERK) and further activate downstream pro-apoptotic genes, leading to cell apoptosis and tissue necrosis, thereby further worsening the host’s infection. Meanwhile, MDV has been previously reported to cause apoptosis (29, 30), but the mechanism remains unclear. Until now, there were no clear and well-investigated route of infection and spread of MDV, which leads us to imagine whether a mechanism similar with ASFV exists in MD, that is, apoptosis was manipulated by MDV, and the cellular debris or apoptotic vesicle produced by apoptotic cells could be phagocytosed and absorbed by adjacent cells to achieve viral delivery, this speculation needs further investigation.

Overall, TNFRSF11B played a crucial role in the MDV infection process as demonstrated in our study. Based on the functional role of TNFRSF11B in MDV infection, it holds promise for future applications in the prevention and control of MD, such as developing small-molecule agonists or recombinant proteins. It also could be incorporated into strategies to enhance vaccine efficacy. However, these potential applications require further in-depth research to substantiate their feasibility.

It should be noted that all the results in the study were obtained from CEF cell models under attenuated MDV strain infection or non-infected conditions. In the future, we need to conduct functional validation in highly virulent strains. The direct interactions between TNFRSF11b and MDV-encoded proteins also need further investigation.

## 5 Conclusion

In conclusion, we reconfirmed that poly I: C pretreatment could reduce MDV infection *in vitro*, and we also identified a candidate gene, TNFRSF11B, which was down-regulated in MDV-infected cells and tissues. The overexpression of TNFRSF11B could inhibit MDV infection, and the knockdown of TNFRSF11B promoted MDV-induced cell apoptosis, and increased MDV infection level.

## Data Availability

All data was shown in the paper in figures or tables or Supplementary files. RNA-seq data is available at NCBI Gene Expression Omnibus (PRJNA1298888).

## Supplementary material

Supplementary Table S1 DEG list in the poly I:C treatment group compared with control group.

Supplementary Figure S1 DEGs enriched in cytokine interaction with its receptor pathway.

Supplementary Figure S2 Knocking down TNFRSF11B promotes apoptosis caused by MDV infection. **(A)** Apoptosis rate of the TNFRSF11B knockdown group compared to the control group at 24 h post MDV infection. **(B)** MDV infected rate (at 24 h post infection) of the TNFRSF11B knockdown group compared to the control group.
