# Supplementary figures and images for: TNFRSF11B modulates Marek’s disease virus infection by regulating apoptosis in chicken embryo fibroblasts
Source: Front Vet Sci. 2026 Feb 5;13:1727743. doi: 10.3389/fvets.2026.1727743 (PMC12916386; doi:10.3389/fvets.2026.1727743)

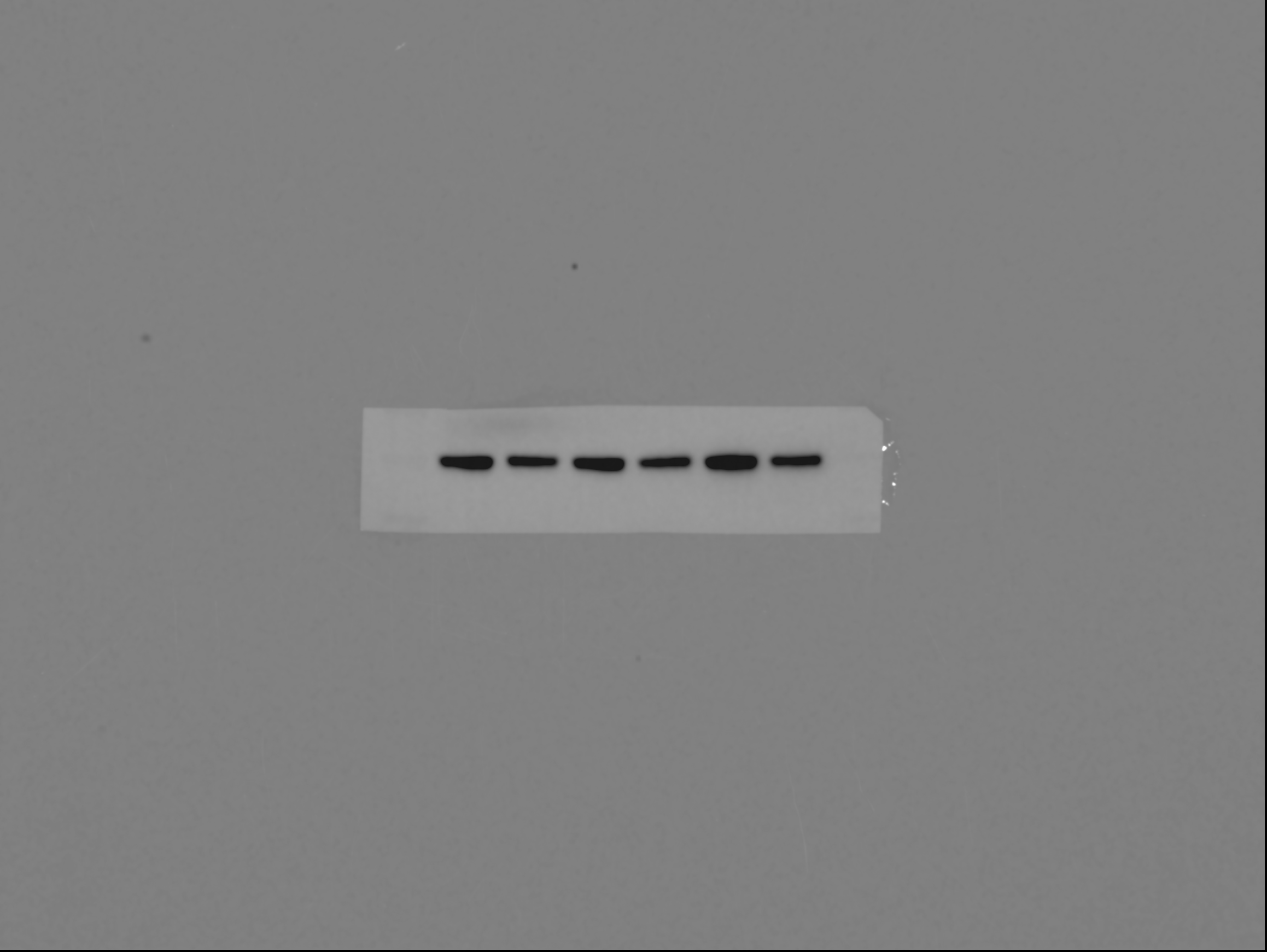

Supplement: Supplementary Figure S1 — DEGs enriched in cytokine interaction with its receptor pathway. [file Image_1.tif]

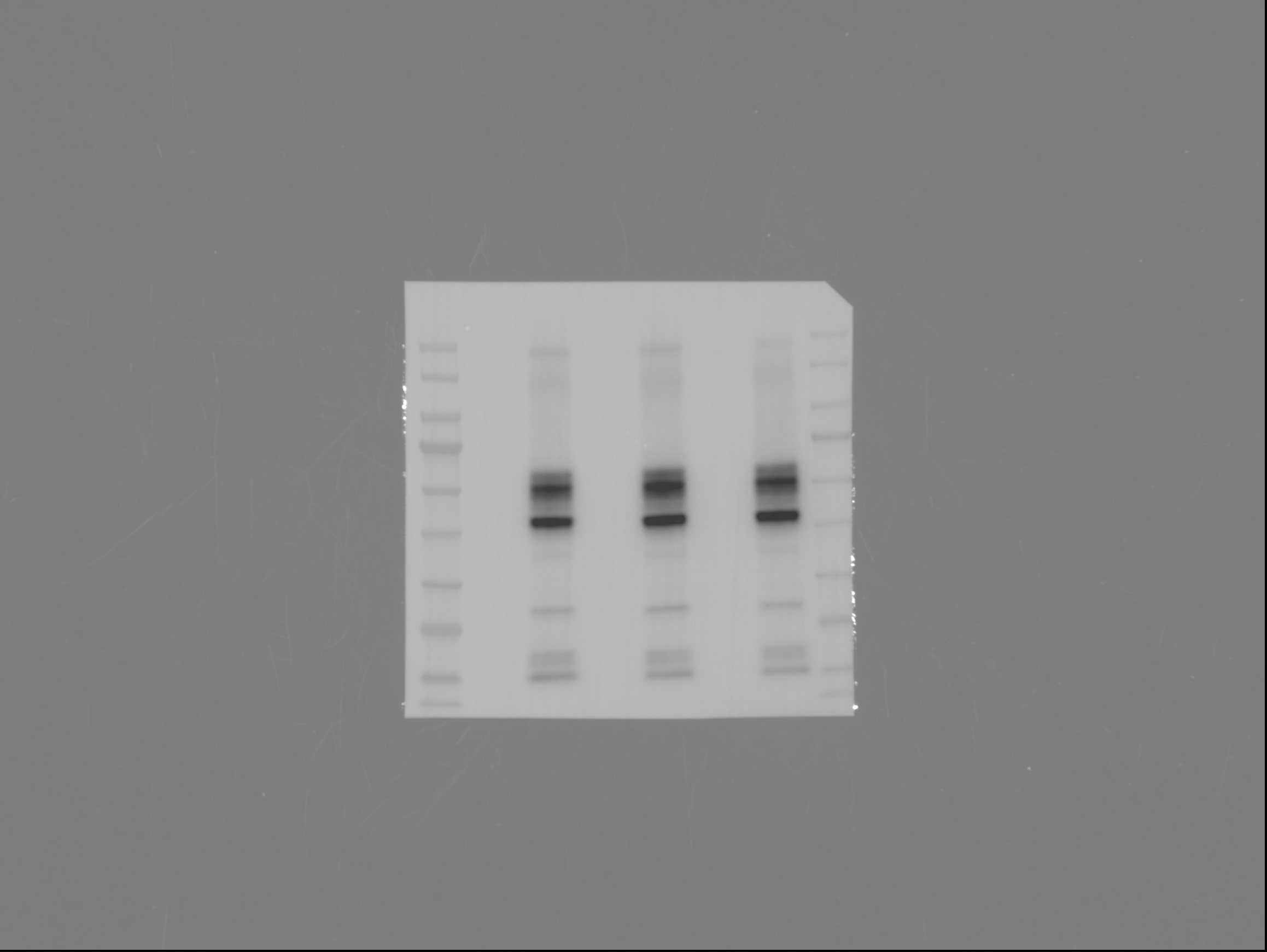

Supplement: Supplementary Figure S2 — Knocking down TNFRSF11B promotes apoptosis caused by MDV infection. (A) Apoptosis rate of the TNFRSF11B knockdown group compared to the control group at 24 h post MDV infection. (B) MDV infected rate (at 24 h post infection) of the TNFRSF11B knockdown group compared to the control group. [file Image_2.tif]
